# The use of folic acid, iron salts and other vitamins by pregnant women in the 2015 Pelotas birth cohort: is there socioeconomic inequality?

**DOI:** 10.1186/s12889-019-7269-0

**Published:** 2019-07-05

**Authors:** Vanessa Iribarrem Avena Miranda, Tatiane da Silva Dal Pizzol, Marysabel Pinto Telis Silveira, Sotero Serrate Mengue, Mariângela Freitas da Silveira, Bárbara Heather Lutz, Andréa Dâmaso Bertoldi

**Affiliations:** 1 Post-Graduate Program in Epidemiology, Federal University of Pelotas (UFPel), Rua Marechal Deodoro, 1160, Centro, Pelotas, RS CEP 96020-220 Brazil; 2 Post-Graduate Program in Epidemiology, Federal University of Porto Alegre, Av. Ipiranga, 2752, sala 203 Porto Alegre, RS – Brasil, Porto Alegre, CEP 90610-000 Brazil; 3 Faculty of Medicine, Maternal and Child Department, Post-Graduate Program in Epidemiology, Federal University of Pelotas, Rua Marechal Deodoro, 1160, Centro, Pelotas, RS CEP 96020-220 Brazil

**Keywords:** Pregnant women, Folic acid, Iron salts, Vitamins, Use medication, Cohort studies

## Abstract

**Background:**

Many low- and middle-income countries recommend micronutrient supplements for pregnant women to improve their nutritional status, prevent possible deficiencies and avoid fetal healgth consequences. This study evaluated the influence of socioeconomic status on the use of folic acid, iron salts and other vitamins and minerals among pregnant women in the 2015 Pelotas Birth Cohort.

**Methods:**

This population-based birth cohort study was carried out with 4270 women. Participants were interviewed during pregnancy and at the maternity hospital about the antenatal period; including the use of iron salts, vitamins and other minerals. Descriptive analyses were performed to characterize the sample. The analyses were adjusted according to socioeconomic variables (maternal education, ethnicity, household income).

**Results:**

The overall prevalence of the use of folic acid, iron salts or other vitamins and minerals was 91.0% (95% CI: 90.1–91.8). Specifically, 70.9% (95% CI: 69.5–72.3) used folic acid, 72.9% (95% CI: 71.5–74.3) used iron compounds, and 31.8% (95% CI: 30.3–33.2) used other vitamins or minerals. In the adjusted analysis, the use of iron salts was associated with nonwhite mothers, with ≤4 years of education and whose family income was less than or equal to the monthly minimum wage. The use of folic acid and other vitamins and minerals was associated with white mothers who were more highly educated and had a higher family income.

**Conclusion:**

Although folic acid and other vitamins and minerals were more frequently used in white, richer and more educated mothers, which indicates inequality, iron supplements were more frequently used in the poorer, less educated nonwhite mothers, suggesting the opposite association for this supplement.

## Background

Micronutrient deficiencies are common among women of reproductive age, and multiple supplements are often indicated for pregnant women. These micronutrient deficiencies may occur due to losses or malabsorption associated with disease, inadequate intake, high fertility rate, short intervals between pregnancies, lack of knowledge about the importance of nutrition during the antenatal period, pregnancy or physiological changes that occur during pregnancy [1–3]. Thus, many low- and middle-income countries recommend micronutrient supplements for pregnant women to improve their nutritional status, prevent possible deficiencies and avoid fetal health consequences [1–5].

Although adequate food intake remains the preferred means of meeting micronutrient requirements, some micronutrient requirements are difficult to meet during pregnancy through diet alone. The most important micronutrients during pregnancy, which are commonly obtained in the form of supplements, include vitamins A, C, D, and E; folic acid; iron; zinc; iodine; copper; selenium; and the B vitamins [2, 4].

Supplementation during the antenatal period can benefit both the mother and fetus [3]. Prophylactic use of iron salts prevents anemia and reduces the risk of low birth weight [6, 7]. Folic acid helps prevent neural tube defects [8]. Iodine can prevent cretinism and aid in fetal growth, as well as reduce the risk of stillbirths, miscarriages and anomalies [1]. Calcium is associated with reduced risks of pre-eclampsia, low birth weight and premature birth [1]. Supplementation with other minerals, such as copper, selenium, magnesium and zinc, has also been shown to reduce labor complications and contribute to fetal development [1, 2, 9].

In response to these needs, some countries fortify selected foods and/or recommend the use of supplements during the antenatal period [6]. The World Health Organization (WHO) strongly recommends daily oral iron and folic acid supplements as part of antenatal care to reduce micronutrient deficiencies. Iron and folic acid supplements should be taken throughout the pregnancy, and the supplementation should begin as soon as possible, regardless of gestational age [6].

In Brazil, although almost all Brazilian pregnant women attend at least one antenatal visit, the proportion of women who attended six or more visits was 73% in 2012, and this percentage was lower in young women with lower socioeconomic status and those who were less educated [10, 11]. Additionally, receiving antenatal care early in a pregnancy is common in only three-quarters of women, with a lower prevalence in women who are younger, black and from the North and Northeast regions of the country [11], consistently pointing out socioeconomic inequalities in antenatal care [11, 12].

Despite the existence of health programs that recommend supplementation as a component of antenatal care, the literature indicates that the use of iron and folic acid is lower than expected [5, 12–14]. Although some studies have reported the use of supplements during pregnancy, few have focused on socioeconomic inequality in their use [12, 15, 16], which is a very important fact, especially in low- and middle-income countries where there are diverse socioeconomic contexts [17].

Thus, the objective of this study was to analyze the influence of socioeconomic status on the use of folic acid, iron salts and other vitamins and minerals during pregnancy.

## Methods

This study is part of the Pelotas Birth Cohort of 2015 (C2015), which was conducted in the city of Pelotas in southern Brazil. All women living in the urban area of Pelotas who gave birth in the city’s five maternity wards between January 1 and December 31, 2015 were invited to participate. More details of the study can be found in the cohort profile paper [18].

Mothers were interviewed at the maternity hospital a few hours after delivery and responded to a standardized questionnaire about the antenatal period, including the use of folic acid, iron salts or other vitamins and minerals. The questionnaire applied was already used in a similar way in other Pelotas birth cohort studies [19–21] and is available on the website of the research center: (http://www.epidemio-ufpel.org.br/uploads/downloads/Perinatal.pdf). In total, 4270 women were recruited for the perinatal study, representing 98.7% of all women in the eligible population [18].

A total of 75% of the women included in the perinatal study were followed up since pregnancy, which allowed us to qualify the information regarding the use of supplements during this period.

The following questions (translated to English) were used for the collection of data regarding supplement use: “Have you used or are you using any vitamin, calcium, folic acid, or iron salt supplements since you became pregnant?” For each supplement reported, the following questions were asked to characterize their usage: “Why do/did you use it?” (routine/prevention, anemia/deficiency, other), “How many days per week do/did you use it?”, “Who told you to use it?” (a doctor or nurse, another person or yourself), and “In which trimester/s did you use this supplement?” (first, second or third).

The supplements were initially classified as folic acid, iron salts or vitamins and other minerals. Those containing iron salts were classified as isolated ferrous sulfate, associations of iron, other isolated forms of iron, and unspecified iron compounds. There was only one subgroup for folic acid due to the recommended daily allowance for pregnant women: folic acid associated with vitamin E. Due to the low dosage, any other forms were classified with the other groups.

The other vitamins and minerals were classified into large groups: calcium carbonate, mineral supplements, multivitamins (including vitamins only), vitamin D, multivitamin and mineral supplements, and other vitamins.

After classifying all supplement groups, it was possible to generate the dependent variables “use of iron salts”, “use of folic acid” and “use of other vitamins and minerals”.

The independent variables that were analyzed were maternal age (collected in complete years and categorized as ≤19, 20–29, 30–46); ethnicity (self-reported by mothers as white, black or other); parity (total number of deliveries, including stillbirths and current pregnancy; later categorized as 1, 2, 3 or 4 or more); mother’s schooling (number of years of study, later categorized into four groups: 0–4, 5–8, 9–11 and 12 or more years) and family income expressed in local currency and converted into a multiple of minimum wage at the time of the perinatal interview (categorized as ≤1, 1.1 to 3.0, 3.1 to 6.0, 6.1 to 10 and > 10). A ‘minimum wage’ is a measure of the legal minimum monthly salary for formal employees in Brazil.

All deliveries were performed in one of five Pelotas maternities (two medical school hospitals, one of them exclusively for users of the Unified Health System (SUS)).

Data analysis was performed using STATA®, version 12.1. The sample was initially described according to the independent variables. Supplement use frequency was then calculated according to the independent variables, yielding the respective confidence intervals (95% CIs).

Crude and adjusted analyses using Poisson regression with robust variance were carried out to verify whether the use of iron, folic acid and other vitamins and minerals differed according to socioeconomic variables. All socioeconomic variables (ethnicity, education and family income) obtained a *p* value < 0.2 in the crude analysis, so they were included in the adjusted analysis. A heterogeneity test and a linear trend test (ordinal variables) were performed. The variables with a p value < 0.05 were considered statistically significant.

To characterize the supplements, a stratified analysis according to gestational trimester was performed. To illustrate the absolute inequality between the use of these supplements according to the trimester of use and family income, we use an equiplot, a graph illustrating the frequency of use of supplements by socioeconomic strata.

This study was approved by the Universidade Federal de Pelotas School of Physical Education Ethics Committee (522.064) and was registered in the National Ministry of Health’s *Plataforma Brasil*. All mothers signed a free and informed consent form before being interviewed.

## Results

Of the 4270 mothers who participated in C2015, 3886 (91.0, 95% CI, 90.1–91.8) reported using folic acid, iron salts or other vitamins and minerals during pregnancy. Of these, 70.9% (95% CI 69.5–72.3) used folic acid, 72.9% (95% CI 71.5–74.3) used some type of iron compound and 30.3% (95% CI 30.3–33.2) used other vitamins or minerals. Table 1 shows the sample characteristics and prevalence of supplement use according to the studied variables. The majority of the mothers were white (70.5%), between 20 and 29 years of age (47.3%), had 9 to 11 years of education (34.3%), had a family income of 1.1–3.0-times the minimum wage (47.2%) and were experiencing their first pregnancy (49.5%).

**Table 1:** Description of mothers participating in the 2015 Pelotas Birth Cohort perinatal study regarding the use of folic acid, iron salts or other vitamins and minerals (*N* = 4270 women)

			Folic acid, iron salts or other vitamins and minerals		
	N	%	N	%	IC 95%
Ethnicity					
White	3005	70.5	2765	92.0	91.0–93.0
Black	680	15.9	607	89.2	86.9–91.6
Other	578	13.5	510	88.2	85.6–90.9
Age (years)					
< =19 years	630	14.7	556	88.3	85.7–90.8
20–29	2021	47.3	1839	91.0	89.7–92.2
30–46	1618	37.9	1490	92.1	90.8–93.4
Education (years)					
0–4 years	394	9.2	321	81.5	77.6–85.3
5–8	1098	25.7	958	87.3	85.3–89.2
9–11	1463	34.3	1346	92.0	90.6–93.4
12 or more	1314	30.8	1261	96.0	94.9–97.0
Family Income (multiple of minimum wage)^a^					
≤ 1	548	12.8	464	84.6	81.6–87.6
1.1–3.0	2015	47.2	1810	89.8	88.5–91.1
3.1–6.0	1126	26.4	1054	93.6	92.2–95.0
6.1–10.0	316	7.4	299	94.6	92.1–97.1
> 10.0	263	6.2	258	98.1	96.4–99.8
Parity					
1	2114	49.5	1988	94.0	93.0–95.0
2	1315	30.8	1206	91.7	90.2–93.2
3	472	11.1	409	86.7	83.6–89.7
4 or more	367	8.6	282	76.8	72.5–81.2
Total	4270	100	3886	91.0	90.1–91.8

^a^ monthly minimum wage in 2015 (R$788 or $201)

Pelotas, RS, Brazil, 2015

The use of folic acid, iron salts or other vitamins and minerals was more frequent in the following groups: white women, women between 30 and 46 years of age, those with 12 years or more of schooling, those with a family income over 10-times the minimum wage and those who were experiencing their first pregnancy. However, the 95% CIs of these prevalence rates overlapped in most categories (Table 1).

Considering the delivery hospitals, 46.4% of deliveries occurred in private hospitals; 35.0% occurred in a school hospital, which also attends private patients and accepts health insurance; and 18.5% occurred in a SUS hospital.

Regarding the characteristics of supplement use according to family income, the majority of supplement use was recommended by doctors or nurses, although iron and other vitamin and mineral supplements were recommended equally for both rich and poor women (approximately 98%). Folic acid was recommended for 99.0% the poorest mothers, compared to 95.0% of the richest mothers. Routine/prevention was the most commonly reported reason for using iron salts, and the reasons were equally distributed between the richest and the poorest mothers. For folic acid and other vitamins and minerals, the most commonly reported reason for use was routine/prevention (92.4 and 69.3%, respectively), differing between rich (97.0 and 74.4%, respectively) and poor mothers (87.0 and 62.3%, respectively). Poor mothers more frequently cited deficiency, while richer mothers reported the usage as routine (data not shown in the table).

**Fig. 1:**
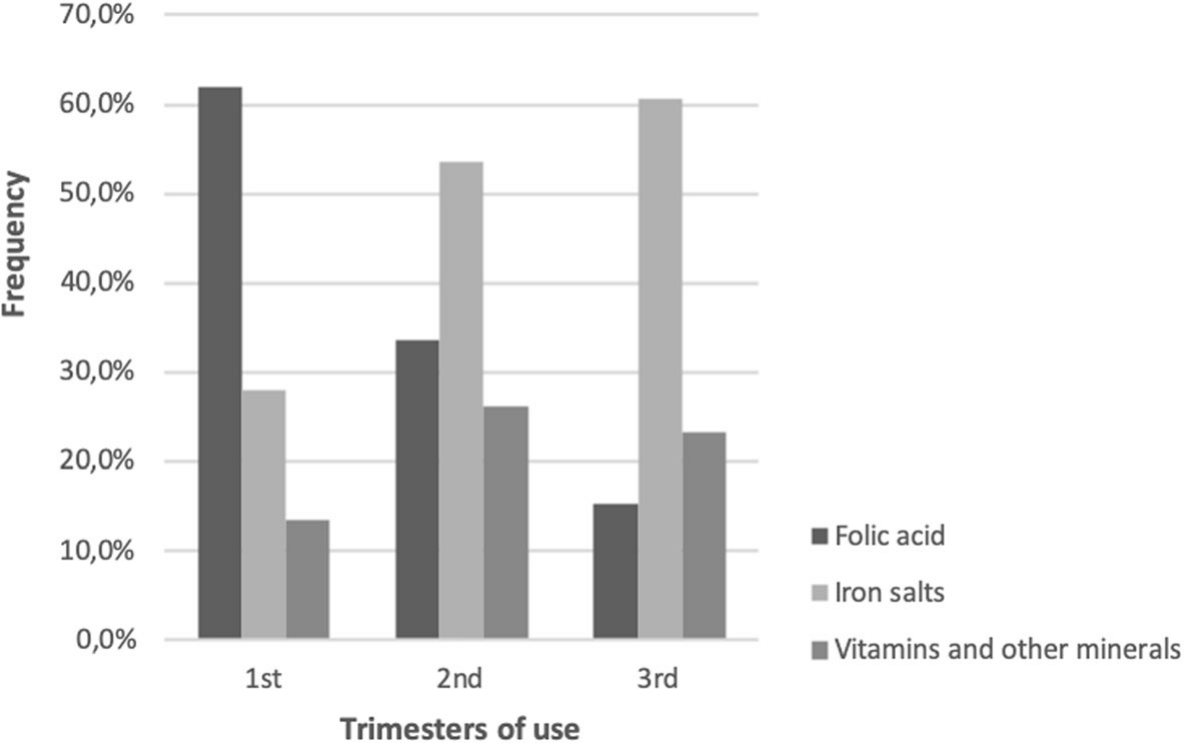
Use of folic acid, iron salts and other vitamins and minerals among pregnant women in the Pelotas Birth Cohort (N = 4270 women). Pelotas, RS, Brazil, 2015

Folic acid use declined closer to delivery, falling from 62.0% in the first trimester (T1) to 15.2% in the third trimester (T3) (Fig. 1). The opposite occurred with iron salts; the use in T3 was 60.7%. The use of the other vitamins and minerals totaled 14.1% of all supplements in T1 and 25.3% in T3. The frequency of the use of the other vitamins and minerals was 13.0% in T1 and 23.2% in T3.

**Fig. 2:**
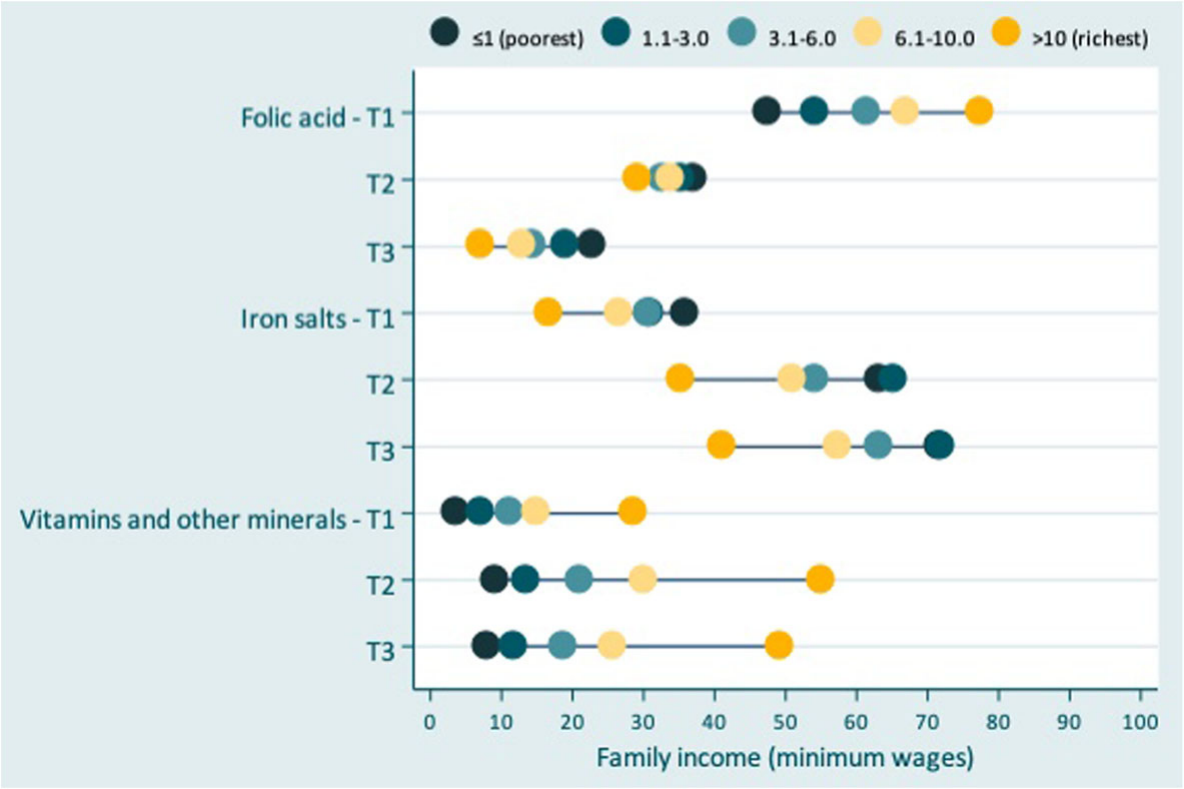
Use of folic acid, iron salts and other vitamins and minerals among pregnant women in the Pelotas Birth Cohort according to the trimester of use and family income (*N* = 4270 women). Pelotas, RS, Brazil, 2015

Figure 2 shows the use of folic acid, iron salts and other vitamins and minerals according to trimester and family income. In the first trimester of pregnancy, folic acid was more frequently used by the richest mothers with family incomes between 6.1–10-times the minimum wage (67.0%) and > 10-times the minimum wage (77.6%). Iron salts were more frequently used in the poorest mothers, regardless of the trimester. Other vitamins and minerals were more frequently used in the richest mothers (> 10-times the minimum wage) during all trimesters (*p* < 0.005 for all values).

Table 2 describes the socioeconomic factors associated with the use of folic acid and iron salts separately. In both the crude and adjusted analyses, all variables were significantly associated with both outcomes. In the adjusted analysis of folic acid use, black mothers used folic acid 9.0% less than white mothers. Women with 4 years or less of education used 22.0% less folic acid than those who were better educated. The use of folic acid was 17.0% lower among mothers with lower family income than among the wealthiest mothers.

**Table 2:** Prevalence of folic acid and iron salt use by pregnant women in the 2015 Pelotas Birth Cohort perinatal study, crude and adjusted prevalence ratios and respective confidence intervals (95% CI) (*N* = 4270 women)

	Iron salts					Folic acid				
	Crude analysis			Adjusted analysis^a^		Crude analysis			Adjusted analysis^a^	
			95% CI		95% CI			95% CI		95% CI
	P (%)	PR	(*p* value)	PR	(*p* value)	P (%)	PR	(*p* value)	PR	(*p* value)
Ethnicity			(< 0.001)		(< 0.001)			(< 0.001)		(0.03)
White	68.2	1		1		73.4	1		1	
Other	85.8	1.25	1.20–1.31	1.11	1.07–1.16	66.6	0.90	0.85–0.96	0.97	0.91–1.03
Black	82.8	1.21	1.15–1.27	1.08	1.03–1.13	62.4	0.85	0.79–0.91	0.91	0.85–0.98
Education (years)			< 0.001		< 0.001			(< 0.001)		(< 0.001)^c^
≤ 4 years	90.6	1.72	1.61–1.83	1.46	1.36–1.56	58.6	0.71	0.65–0.79	0.78	0.71–0.86
5–8	87.4	1.66	1.56–1.75	1.45	1.35–1.53	62.7	0.77	0.72–0.81	0.82	0.77–0.87
9–11	77.3	1.46	1.38–1.56	1.31	1.23–1.40	69.9	0.86	0.82–0.89	0.88	0.84–0.93
≥ 12	52.6	1		1		81.3	1		1	
Family Income ^b^			(< 0.001)		(< 0.001)			(< 0.001)		(0.02) ^c^
≤ 1	86.4	1.93	1.68–2.23	1.39	1.20–1.61	57.9	0.68	0.61–0.74	0.82	0.74–0.90
1.1–3.0	80.7	1.81	1.57–2.07	1.39	1.20–1.59	68.3	0.80	0.75–0.85	0.91	0.85–0,97
3.1–6.0	67.3	1.50	1.30–1.74	1.28	1.11–1.48	75.8	0.88	0.83–0.94	0.95	0.89–1.01
6.1–10.0	48.8	1.10	0.91–1.31	1.06	0.89–1.26	77.6	0.90	0.84–0.98	0.92	0.85–1.00
> 10.0	44.6	1		1		85.3	1		1	

^a^ Model adjusted for ethnicity, family income and years of education

^b^ Monthly minimum wage in 2015 (R$788 or $201)

^c^ *p*-value: χ^2^ test for trend

Pelotas, RS, Brazil, 2015

Nonwhite mothers used up to 8.0% more iron salts than white mothers. Women with ≤4 years of schooling used 46.0% more iron salts than those with more education. In addition, those with a family income ≤1-times the minimum wage used iron more frequently (39.0%).

The adjusted analysis of other vitamins and minerals showed that mothers who were white, more educated and had higher incomes used more vitamins and minerals than any other group (Table 3).

**Table 3:** Prevalence of the use of other vitamins and minerals by pregnant women in the 2015 Pelotas Birth Cohort perinatal study, crude and adjusted prevalence ratios and respective confidence intervals (95% CI) (*N* = 4270 women)

	Crude analysis				Adjusted analysis^a^		
	P (%)	PR	95% CI	(*p* value)	PR	95% CI	(*p* value)
Ethnicity				< 0.001			< 0.001
White	62.7	1			1		
Other	18.3	0.49	0.41–0.58		0.77	0.65–0.91	
Black	17.8	0.48	0.39–0.58		0.73	0.61–0.86	
Education (years)				< 0.001			< 0.001
≤ 4 years	6.9	1			1		
5–8	11.7	1.70	1.10–2.64		1.50	0.97–2.33	
9–11	26.0	3.80	2.50–5.73		2.91	1.91–4.43	
≥ 12	59.6	8.70	5.79–13.0		5.22	3.44–7.93	
Family Income ^b^				< 0.001			< 0.001
≤ 1	10.8	1			1		
1.1–3.0	20.9	1.94	1.47–2.56		1.37	1.04–1.80	
3.1–6.0	40.2	3.73	2.84–4.90		1.86	1.41–2.44	
6.1–10.0	67.2	6.23	4.74–8.20		2.37	1.78–3.15	
> 10.0	70.2	6.51	4.95–8.55		2.35	1.76–3.10	

^a^ Model adjusted for ethnicity, family income and years of education

^b^ Monthly minimum wage in 2015 (R$788 or $201)

Pelotas, RS, Brazil, 2015

## Discussion

The findings of the present study were a 91% overall prevalence of supplement use, a result similar to those obtained by Forster et al. in Australia [5] and Bagheri et al. in Iran [22], who found that 92.7% of the women used some multivitamin during pregnancy. However, the majority of similar studies have found lower prevalences, ranging from 70.3 to 88.7% [23–26].

The prevalence of folic acid and iron compound use was 70.9 and 72.9%, respectively. The use of these supplements during pregnancy is advocated by both the WHO and the Brazilian Ministry of Health, which recommend the use of these supplements for all pregnant women as part of antenatal care [6, 27]. An Australian study [5] of women in the immediate postpartum period revealed iron and folic acid use of 52.0 and 79.0%, respectively, during pregnancy, while a study from Portugal found prevalence rates of 55.4 and 81.9% for iron and folic acid use, respectively [26]. On the other hand, an Italian cohort study of 1000 women found a prevalence of iron salt use of 95.0% [28]. Lower prevalences of folic acid use, approximately 30.0%, were found by Mezzomo, et al. [29] and Barbosa et al. [14] in Brazil.

With respect to iron supplementation, these differences could be attributable to different social contexts and regional variations in recommendations, a factor especially present in low- and middle-income countries where dietary iron intake is often insufficient [15, 17]. Regarding the trend of iron use according to the trimester of pregnancy, the increase in use in the third trimester may be due to the fact that many mothers discover pregnancy and begin antenatal care with the most advanced pregnancy, thus not having access to the recommendation of use in the first and/or second trimester of pregnancy. In addition, in the third trimester there are more pregnant women with iron deficiency anemia, which increases adherence to the recommendations of using iron salts [27, 30, 31].

Differences in folic acid use could be due to differences between protocols. During the study period, the current protocol in Brazil recommended the use of folic acid in the pre-conception and during the first trimester only [27]. However a more recent protocol recommends the use of folic acid during all period of pregnancy [32]. In addition, this supplement is often not prescribed when a woman’s antenatal care begins at a late stage. However, for women seeking preconception counseling, there is a high chance that it will be prescribed for the all gestational period.

So, despite the fact that iron and folic acid are commonly prescribed in most countries, in addition to the reasons mentioned above, nonuse by pregnant women persists for others variety of reasons. For iron, for example, these include adverse reactions and ignorance of its importance [15]. There is also controversy in the literature about the benefits and possible side effects of iron use by non-anemic mothers [33, 34].

Consistent with the literature, we found that the use of supplements in general was more prevalent among white women [29, 35], older women [34, 35], those with a higher education level [9, 14, 29] and those with a higher income [15, 24, 29]. Although these characteristics suggest some inequality, their differences were not significant. Multiparous women used fewer supplements than those experiencing their first pregnancy. This difference is perhaps due to greater concern for the first child, which diminishes somewhat with subsequent pregnancies, given that prior experience and changes in attitudes about health make the mother less insecure [15, 36].

The characteristics of folic acid, iron salt and other vitamin and mineral use indicate that practically all supplements used during antenatal care were recommended by doctors [16, 25]. Regarding the different reasons for use, 49.8% of women in the iron salts group were treating a deficiency, while for folic acid and other vitamins and minerals, the most frequently reported reason was routine prevention. This difference could be due to increased clinical and laboratory monitoring of iron deficiency anemia compared with that of other micronutrient deficiencies.

The results for the use of each supplement according to trimester and family income are in agreement with the findings of the stratified analysis. Regarding iron salts, the results showed that the nonwhite, poorer and less educated mothers used them more, which would seem to suggest that iron supplementation is more frequent among poorer and nutritionally vulnerable women going in the same direction of the results pointed out by the study of Tomasi et al. 2017, which included all the basic health units in Brazil and found prevalence of iron prescriptions of almost 100% among women with the same characteristics [12]. However the opposite has been found in other studies [15, 16], and this difference may be related to the fact that, in Brazil, prenatal coverage is considered high, and women who use public health services the most are the poorest.

Unlike the use of iron salts, the use of folic acid and other vitamins and minerals reflected different user profiles, i.e., white, wealthier and more educated [9, 13–16, 26, 35]. This difference could be due to the fact that poorer mothers seek health services later in pregnancy when folic acid would no longer be recommended, whereas mothers with better purchasing power and greater access to information would tend to have a planned pregnancy and, thus, a greater chance of beginning antenatal care (and supplementation) early in T1 [15, 16].

Our study has the advantage of being a cross-sectional study nested in a population-based cohort, which provides more timely information about supplement use. However, it also has some limitations. No information regarding dose or adherence was analyzed. However, the fact that iron salts, vitamins and other minerals were used on average 6.9 days per week can be considered a proxy for the continuity of treatment.

The elapsed time between supplement use and the interview can lead to recall error and underestimation of prevalence. Nevertheless, a recent study demonstrated good agreement between short recall periods for continuously used drugs [37]. In addition, the supplement use data collected during antenatal care minimizes this problem.

Since part of the sample did not participate in the antenatal study, subgroup analyses were conducted. The results indicated no differences in the prevalence of iron salt use among those who participated only in the perinatal study. However, there was a lower prevalence of the use of other compounds in this group. Thus, there may have been an underestimation of the general use of folic acid, iron salts and other vitamins and minerals when analyzed separately.

## Conclusion

This exploration of socioeconomic differences in the use of supplements found inequalities in the use of folic acid and other vitamins and minerals in favor of the richer and more educated white mothers, but in the case of vitamins and other minerals, there are no recommendations for their use or evidence of well-established benefits in the pregnant population.

However, in the case of folic acid, this inequality is relevant since it is universally recommended and has well-established benefits. Regarding the use of iron salts, the opposite occurred: an inequality favored nonwhite, less educated and poorer mothers. This suggests equity in access and demonstrates the reach of this important public health policy.

Thus, it is concluded that the existing recommendations are sufficient, however, it is necessary that there is an incentive to the early initiation of antenatal care, promotion of the knowledge of the pregnant women and the health professionals of the area on the importance of the use of the folic acid and iron salts in the gestational period, in order to increase the adherence to the recommendations.

## Data Availability

The datasets generated and/or analyzed during the current study are not publicly available because they are extremely long and costly, both in terms of the money and time involved in project writing and data collection, which involves complex data and local specificities. Thus, responsible use of the data includes knowledge of the study design and objectives and of the local health system. However, data can be made available by the 2015 cohort team coordinator on reasonable request and agreement.

## Abbreviations

C2015: Pelotas Birth Cohort of 2015
CI: Confidence interval
T1: First trimester
T2: Second trimester
T3: Third trimester
WHO: World Health Organization
